# Social media addiction relationship with academic engagement in university students: The mediator role of self-esteem, depression, and anxiety

**DOI:** 10.1016/j.heliyon.2024.e24384

**Published:** 2024-01-09

**Authors:** Miguel Landa-Blanco, Yarell Reyes García, Ana Lucía Landa-Blanco, Antonio Cortés-Ramos, Eddy Paz-Maldonado

**Affiliations:** a Degree in Clinical Psychology, School of Psychological Sciences, National Autonomous University of Honduras, Tegucigalpa, Honduras; b Department of Developmental Psychology and Education, Faculty of Psychology and Speech Therapy, University of Malaga, 29010, Malaga, Spain; c Department of Pedagogy and Educational Sciences, National Autonomous University of Honduras, Tegucigalpa, Honduras

**Keywords:** Academic engagement, Social media, Mental health, Depression, Anxiety, Self-esteem, Higher education

## Abstract

This research analyzed how addiction to social media relates to academic engagement in university students, considering the mediating role of self-esteem, symptoms of depression, and anxiety. A quantitative methodology was used with a non-experimental-relational design. A set of questionnaires was applied to a non-probabilistic sample of 412 students enrolled at the National Autonomous University of Honduras. On average, participants use 4.83 different social media platforms at least once a week. Instagram and TikTok users report significantly higher levels of social media addiction, symptoms of depression, and anxiety compared to non-users. Directly, social media addiction does not significantly influence academic engagement scores. However, there are significant indirect inverse effects on academic engagement. Symptoms of depression and self-esteem mediate these effects. Social media addiction increases symptoms of depression, which in turn decreases academic engagement scores. Social media addiction decreases self-esteem, which serves as a variable that significantly increases academic engagement. Overall, findings suggest that social media addiction has a total inverse effect on academic engagement; symptoms of depression and self-esteem mediate this relationship. The implications of these findings are discussed.

## 1 Introduction

### 1.1 Social media addiction

The use of technology has emerged as a significant concern within modern psychology, particularly regarding the potential effects of social media consumption, which can be both harmful and beneficial. Social media platforms are virtual spaces on the Internet facilitating real-time interaction, connection, and communication. Social media has become an integral aspect of human lives, supplanting traditional social practices and transforming the landscape of interpersonal communication [1].

These spaces also allow individuals to shape their identity and tailor the information they receive based on their preferences [2,3]. Social media can enhance people's social capital [4], positively impacting their psychological well-being [5]. Additionally, such platforms help raise public awareness of various mental health issues [6].

However, extended and excessive engagement with social media can lead to a form of addictive behavior. In such a case, many individuals risk developing an obsessive need to stay online, which interferes with their daily lives [7]. This addiction often results in the individual overlooking fundamental necessities such as sleep, personal interactions with loved ones, and daily tasks. It also manifests in various adverse emotional and behavioral states, including interpersonal conflicts [[8], [9], [10]]. Social media addiction has been found to induce effects akin to the consumption of psychoactive substances, contributing to the deterioration of self-esteem, mental health, and academic performance, as demonstrated by several studies [[11], [12], [13]]. Furthermore, individuals tend to underestimate the time they spend on social media, and abstaining from it may cause restlessness, irritability, and withdrawal symptoms [1].

### 1.2 Self-esteem, depression and anxiety symptoms

Self-esteem, a subjective evaluation of an individual's self-worth, can significantly impact well-being and performance [14,15]. It is important to note that self-esteem is not necessarily an objective reflection of a person or how others evaluate them [16]. Greater use of social media has been consistently associated with lower self-esteem scores [17], which, in turn, is correlated with decreased academic performance [18]. This link between social media use, self-esteem, and academic performance underscores the far-reaching consequences of social media addiction on individuals' lives.

In this line, addictive internet use is also linked to increased depressive and anxiety symptoms [17]. Depression is a negative result of the inability to cope with the stresses of life; its symptoms involve persistent and intense feelings of helplessness, despair, discontent, sadness, pessimism, and worthlessness [19]. These symptoms are associated with adverse effects on emotions, thoughts, motivations, social relationships, and physical well-being [20]. Depression symptoms are a prevalent problem faced by many students [21].

On the other hand, anxiety is a reaction with cognitive, psychological, and behavioral components; it is characterized by physical and mental agitation [22]. Emerging research suggests that social media addiction exerts a multifaceted influence on students' academic performance and psychological well-being. While its impact on academic performance is indirect, it directly contributes to elevated stress and anxiety levels among students. This heightened stress can further exacerbate anxiety, which in turn negatively impacts academic performance. Additionally, stress directly contributes to anxiety, potentially leading to depression. These findings underscore the significance of social media addiction and its potential substantial impact on students' psychological well-being and academic trajectories [23].

### 1.3 Academic engagement

Academic engagement encompasses the extent to which students actively participate in and are involved in pedagogical activities within their formal education. Academic engagement emerges as a pivotal factor in evaluating student well-being. Numerous scientific studies delve into this matter from diverse perspectives, encompassing institutional factors, individual aspects, basic psychological needs, legal vulnerability, higher education absenteeism, and university students' employability [[24], [25], [26], [27]].

Academic engagement correlates with successful academic performance and the integration of students into the university environment [28,29]. High engagement fosters academic achievement, facilitates positive adjustment, and enhances students' physical and mental well-being while reducing the likelihood of educational abandonment [30].

Academic engagement can be influenced by various factors, including the student's characteristics, the teaching philosophy, the methods employed by the educators, the student-teacher relationships, and the overall educational context in which the pedagogical practices are implemented [31]. Students with high self-esteem tend to trust their abilities more, increasing their motivation to study, which leads to better academic performance. Consequently, a positive relationship exists between self-esteem, academic engagement, and academic performance [30,32]. However, research indicates that social media addiction may adversely affect mental health, academic performance, and career outcomes [33,34]. Other studies indicate that abusive social media consumption is linked to higher procrastination and distractibility and a decline in productivity, negatively impacting overall academic performance [32].

### 1.4 The context of the COVID-19 pandemic

The COVID-19 pandemic had significant effects on Hondurans' mental health, with respondents reporting high levels of obsessive-compulsive symptoms, anxiety, and interpersonal sensitivity [35]. The pandemic also triggered a series of health restrictions that limited spaces for social interaction and increased academic virtualization. As a result, there was a significant increase in the use of social media, an issue that, if neglected, has a harmful potential on people's well-being [36,37].

A meta-analysis examining behavioral addictions during the COVID-19 pandemic revealed that individuals under lockdown were twice as likely to develop social media addiction when compared to non-lockdown periods. This analysis also demonstrated that younger individuals were at an increased risk of social media addiction. Additionally, national-level data indicated that social media addiction was 52.5 % higher in developed countries compared to developing countries [38].

On the other hand, a study conducted among undergraduate students in Saudi Arabia during the COVID-19 pandemic found that problematic social media use significantly leads to increased technostress and exhaustion, negatively impacting academic performance. The study identified technostress and exhaustion as pivotal mediators in the relationship between problematic social media usage and academic performance [39].

### 1.5 The present study

**Fig. 1:**
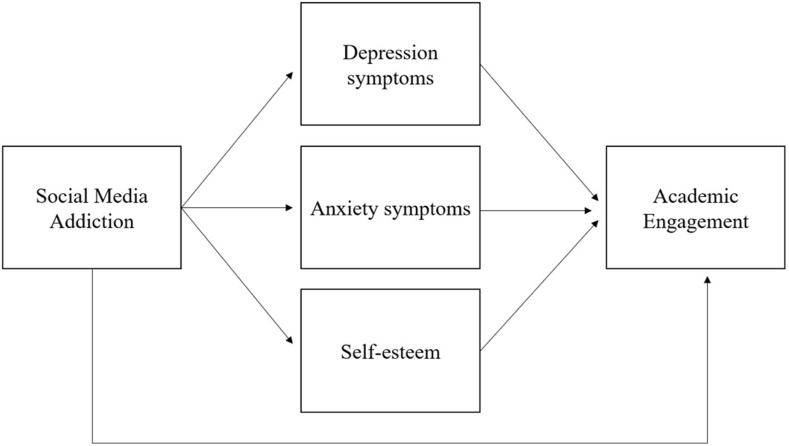
Hypothetical mediation model.

This research aims to analyze addiction to social networks and its impact on academic engagement, considering the possible mediating role of self-esteem, depression, and anxiety symptoms. These variables are framed in a population of students enrolled at the National Autonomous University of Honduras, the country's largest university. Previous research suggests this population is at particular risk of mental health and academic problems [40,41], a situation that highlights the importance of the current study. Additionally, recent research indicates that there still is a critical knowledge gap regarding the problematic use of social media [9]. Data was collected in August 2022. The study's hypotheses and their respective rationale are presented in Table 1 and Fig. 1.

**Table 1:** Hypotheses of the study.

Hypothetic relationship between variables	Rationale
Hypothesis 1: Social media users will report worse mental health outcomes than non-users.	Previous studies have found that social media consumption may increase symptoms of depression and anxiety [42], and decrease self-esteem [43].
Hypothesis 2: Social media addiction ⇒ depression⇒ academic engagement	Students with higher scores on social media addiction report higher depressive symptomatology [8]. Previous research suggests that symptoms of depression mediate the relationship between mobile phone use and academic performance [44].
Hypothesis 3: Social media addiction ⇒ anxiety⇒ academic engagement	Social media addiction is positively related to higher anxiety symptoms [45]. Concurrently, anxiety has detrimental effects on university students' academic engagement [46].
Hypothesis 4: Social media addiction ⇒ self-esteem ⇒ academic engagement	Previous studies concluded that addictive social media consumption negatively affects self-esteem [47]. Additionally, self-esteem is essential to academic engagement [30].
Hypothesis 5: Social media addiction⇒ academic engagement	Recent research has found a direct inverse relationship between social media addiction and academic engagement; thus, a higher social media addiction is associated with lower academic engagement [48].

## 2 Materials and methods

### 2.1 Participants

In August 2022, when the data was collected, there were a total of 62,082 students enrolled in the National Autonomous University of Honduras (UNAH). Given the population size, at a 95 % confidence interval and a 5 % margin of error, the a priori minimum sample size was estimated at 382 participants. However, the final sample size exceeds the minimum required, as 412 respondents completed the survey. Their ages varied between 18 and 43 years (*M* = 22.22; *SD* = 4.42). Most participants were women (*n* = 269; 65.29 %), with men accounting for 34.71 % (*n* = 143) of the sample. Respondents were selected through a non-probabilistic sampling method based on voluntary participation and snowball strategies. A link to the online survey was shared with teachers who were asked to share it with their students. Additionally, students who completed the questionnaire were asked to recruit new participants. Two filters were included as inclusion criteria: 1) being enrolled at the National Autonomous University of Honduras, and 2) being over 18 years old.

### 2.2 Data collection techniques

#### 2.2.1 Utrecht Work Engagement Scale-Student Version

Academic engagement was measured using the Spanish version of the Utrecht Work Engagement Scale-Student Version (UWES-9S) [49,50]. The UWES-9S is a 9-item, Likert-type questionnaire with a 7-point response set (0 = “never”; 6 = “always”). Total summative scores range between 0 (low engagement) to 54 (high engagement). Based on data from the current study, the UWES-9S achieved an adequate internal consistency, with a Cronbach's alpha of 0.92, *CI* 95 % [0.90; 0.93].

#### 2.2.2 Bergen Social Media Addiction Scale

The Bergen Social Media Addiction Scale (BSMAS) Spanish version contains six items [51,52]. The BSMAS uses a 5-point Likert-type response set (1 = “never”; 5 = “always”). Therefore, summative total scores range between 6 (low social media addiction) and 30 (high social media addiction). Based on data from the current study, the BSMAS achieved an adequate internal consistency, with a Cronbach's alpha of 0.84, *CI* 95 % [0.81; 0.86].

#### 2.2.3 Patient Health Questionnaire

The Spanish version of the Patient Health Questionnaire (PHQ-9) is a 9-item Likert-type scale that measures symptoms of depression experienced during the last two weeks [53,54]. Each item is scored on a scale from 0 (“not at all”) to 3 (“nearly every day”). The sum of the responses to these nine items created a total score ranging from 0 (low symptomatic presence) to 27 (high symptomatic presence), classified as: low (0–4), mild (5–9), moderate (10–14), moderately-severe (15–19) and severe symptoms (20–27) [53]. Based on data from the current study, the PHQ-9 achieved an adequate internal consistency, with a Cronbach's alpha of 0.89, *CI* 95 % [0.88; 0.91].

#### 2.2.4 Generalized Anxiety Disorder

The Spanish version of the Generalized Anxiety Disorder (GAD-7) is a 7-item Likert-type scale that uses a 4-point scoring system (0 = "not at all”; 3 = "nearly every day”). The GAD-7 measures the prevalence of symptoms of anxiety experienced during the last two weeks [55,56]. Total scores range from 0 (low symptomatic presence) to 21 (high symptomatic presence), with scores higher than 10 considered to be of clinical significance [55]. Based on data from the current study, the GAD-7 achieved an adequate internal consistency, with a Cronbach's alpha of 0.90, *CI* 95 % [0.88; 0.92].

#### 2.2.5 Rosenberg Self-Esteem Scale

The Spanish version of the Rosenberg Self-Esteem Scale (RSE) consists of 10 items scored on a 4-point Likert-type scale (1 = “strongly agree”; 4 = “strongly disagree”) [14,57]. Total scores range from 10 (low self-esteem) to 40 (high self-esteem). Based on data from the current study, the RSE achieved an adequate internal consistency, with a Cronbach's alpha of 0.84, *CI* % [0.81, 0.86].

#### 2.2.6 General Data Questionnaire

A closed-ended questionnaire collected general information from the participants, including their sex and age. The respondents were given a list of various social media platforms and asked to identify which ones they use at least once a week. This method enabled the researchers to differentiate between individuals who use specific social media platforms and those who do not (users vs non-users).

### 2.3 Data analyses

First, the internal reliability of each questionnaire was determined using Cronbach's alpha; Confidence Intervals (*CI*) were calculated at a 95 % confidence level. The summative total scores were calculated for each questionnaire. Each variable was analyzed using descriptive statistics, including: mean scores (*M*), standard deviations (*SD*), and relative and absolute frequencies. Then, to test Hypothesis 1, an independent sample Student's *t*-test analysis was made to compare variable scores regarding participants' social media consumption (users and non-users); Cohen's d was used to estimate the effect size of these differences. The relationship between variables was tested using Pearson's *r* coefficient. Finally, a mediation analysis was used to test Hypothesis 2 through Hypothesis 5. All statistics were tested at a 95 % confidence level. The statistical analysis was run in JASP [58].

### 2.4 Ethical considerations

The research followed the ethical guidelines established by the Master's Degree in Clinical Psychology of the National Autonomous University of Honduras (UNAH) and the Code of Ethics of the Psychology Professional in Honduras. Of the latter, the requirements set out in Chapter VII, Articles 55 to 62 were considered. Based on this, informed consent was presented to potential participants. This electronic document detailed the study's title and the authors' institutional affiliation, described the study's purpose, the activities to be completed, the anonymous nature of the information, the subsequent use of the data, and contact information. Accepting the conditions of informed consent was a requirement to access the study. At the end of the survey, participants were presented with a link to “La UNAH Te Escucha”, a free psychological platform that provides real-time psychological attention for those who request it.

The research project was officially submitted to the Committee on Research Ethics (CRE) affiliated with the Faculty of Social Sciences at the National Autonomous University of Honduras. The CRE granted an Exemption Letter based on several factors, including 1) the study's observational design, 2) the anonymous data collection process, 3) the involvement of consenting individuals in the research, and 4) the low-risk nature of the study.

## 3 Results

The most used social media platforms were WhatsApp, Facebook, YouTube and Instagram. TikTok, Pinterest, Snapchat, and Reddit were used less, see Table 2. On average, participants use 4.83 (*SD* = 1.57) social media platforms at least once a week.

**Table 2:** Social media platforms used by the respondents.

Social media	User	Non-user
WhatsApp	374 (90.78 %)	38 (9.22 %)
Facebook	336 (81.55 %)	76 (18.45 %)
YouTube	317 (76.94 %)	95 (23.06 %)
Instagram	286 (69.42 %)	126 (30.58 %)
TikTok	191 (46.35 %)	221 (53.64 %)
Pinterest	122 (29.61 %)	290 (70.39 %)
Snapchat	81 (19.66 %)	331 (80.34 %)
Reddit	25 (6.07 %)	387 (93.93 %)

*Note.* Respondents who reported using the platform at least once a week are considered “users".

Compared to non-users, participants who use Instagram and TikTok report significantly higher levels of social media addiction and symptoms of depression and anxiety (*p* < 0.05). Similarly, Pinterest users report greater symptoms of depression and anxiety than non-users of the platform. Neither academic engagement nor self-esteem varied significantly between users and non-users of specific social media platforms, see Table 3.

**Table 3:** Variable comparisons between users and non-users of specific social media platforms.

Variable	Condition	1 *M (SD)*	2 *M (SD)*	3 *M (SD)*	4 *M (SD)*	5 *M (SD)*	6 *M (SD)*	7 *M (SD)*	8 *M (SD)*
Social media addiction	User	15.35 (5.66)	15.93 (5.63)	15.28 (5.59)	15.20 (5.62)	15.57 (5.54)	16.07 (5.31)	15.36 (6.24)	15.36 (5.16)
	Non-user	14.43 (5.52)	13.46 (5.29)	14.18 (6.07)	15.11 (5.74)	15.01 (5.68)	14.07 (5.74)	15.17 (5.61)	15.13 (5.76)
	Contrast	t = 1.27; p = 0.20; d = −0.16	**t** = **-4.18; p** < **0.001; d** = **-0.45**	t = −1.14; p = 0.26; d = −0.19	t = −0.14; p = 0.89; d = −0.02	t = 0.91; p = 0.37; d = −0.10	**t** = **-4.39; p** < **0.001; d** = **-0.43**	t = −0.17; p = 0.87; d = −0.03	t = −0.32; p = 0.75; d = .0.04
Symptoms of depression	User	12.74 (6.99)	13.20 (7.03)	12.70 (7.08)	12.56 (7.12)	13.83 (7.42)	13.52 (7.29)	12.60 (5.98)	12.48 (6.54)
	Non-user	11.61 (7.49)	10.99 (7.02)	10.82 (7.02)	12.41 (7.04)	11.98 (6.89)	11.67 (6.81)	12.52 (7.16)	12.54 (7.23)
	Contrast	t = −1.26; p = 0.21; d = −0.16	**t** = **-2.94; p** < **.01; d** = **-0.32**	t = −1.56; p = 0.12; d = −0.27	t = −0.18; p = 0.86; d = −0.02	**t** = **2.43; p** = **0.02; d** = **-0.26**	**t** = **-2.67; p** < **.01; d** = **-0.26**	t = −0.05; p = 0.96; d = −0.01	t = 0.06; p = 0.95; d = 0.01
Symptoms of anxiety	User	10.55 (5.63)	11.07 (5.57)	10.57 (5.72)	10.27 (5.63)	11.89 (5.67)	11.62 (5.59)	9.28 (5.51)	10.57 (5.32)
	Non-user	10.59 (5.82)	9.39 (5.72)	10.395 (5.160)	11.48 (5.69)	9.99 (5.57)	9.63 (5.57)	10.64 (5.67)	10.55 (5.75)
	Contrast	t = 0.067; p = 0.95; d = 0.01	**t** = **-2.80; p** < **.01; d** = **-0.30**	t = −0.18; p = 0.86; d = −0.03	t = 1.83; p = 0.07; d = 0.21	**t** = **3.15; p** < **.01; d** = **-0.34**	**t** = **-3.60; p** < **0.001; d** = **-0.36**	t = 1.16; p = 0.25; d = 0.24	t = −0.03; p = 0.95; d = 0.01
Self-esteem	User	26.88 (6.72)	26.55 (6.61)	26.91 (6.68)	27.17 (6.65)	26.45 (6.83)	26.39 (6.58)	27.96 (6.03)	27.95 (6.45)
	Non-user	27.30 (6.41)	27.88 (6.69)	27.40 (6.55)	26.25 (6.67)	27.17 (6.58)	27.45 (6.71)	26.89 (6.70)	26.71 (6.70)
	Contrast	t = 0.50; p = 0.62; d = 0.06	t = 1.88; p = 0.06; d = 0.20	t = 0.43; p = 0.67; d = 0.07	t = −1.18; p = 0.24; d = −0.14	t = 1.00; p = 0.32; d = 0.19	t = 1.62; p = 0.11; d = 0.16	t = −0.78; p = 0.44; d = −0.16	t = −1.50; p = 0.13; d = −0.19
Academic engagement	User	32.32 (11.45)	31.68 (11.54)	32.21 (11.54)	32.35 (11.45)	31.65 (10.95)	32.14 (11.34)	29.84 (12.87)	33.78 (10.63)
	Non-user	32.13 (12.13)	33.68 (11.54)	33.11 (11.98)	32.10 (12.00)	32.56 (11.82)	32.42 (11.78)	32.45 (11.48)	31.92 (11.77)
	Contrast	t = −0.13; p = 0.90; d = −0.02	t = 1.62; p = 0.11; d = 0.17	t = 0.46; d = 0.65; d = 0.08	t = −0.19; p = 0.85; d = −0.02	t = 0.73; p = 0.47; d = 0.08	t = 0.24; p = 0.81; d = 0.02	t = 1.09; p = 0.28; d = 0.23	t = −1.29; p = 0.20; d = −0.16

*Note.* 1 = Facebook, 2 = Instagram, 3 = WhatsApp, 4 = YouTube, 5 = Pinterest, 6 = TikTok, 7 = Reddit, 8 = Snapchat. Significant differences (*p* < 0.05) are presented in bold letters.

Academic engagement is positively correlated with self-esteem (*r* = 0.33), while maintaining an inverse relationship with symptoms of depression (*r* = −0.34), anxiety (*r* = −0.23), and addiction to social media (*r* = −0.10). At the same time, addiction to social media is positively associated with symptoms of depression (*r* = 0.44) and anxiety (*r* = 0.42), while inversely related to self-esteem (*r* = −0.29), see Table 4.

**Table 4:** Bivariate correlations between variables.

Variable	Self-esteem	Social media addiction	Symptoms of depression	Symptoms of anxiety	Academic engagement
Self-esteem	–				
Social media addiction	−0.29***	–			
Symptoms of depression	−0.58***	0.44***	–		
Symptoms of anxiety	−0.49***	0.42***	0.78***	–	
Academic engagement	0.33***	−0.10*	−0.34***	−0.23***	–

*Note.* Bivariate correlations were assessed through Pearson's *r* coefficient.

*p < 0.05, **p < 0.01, ***p < 0.001.

Based on the mediation analysis, social media addiction does not directly affect academic engagement scores (*β* = 0.06, *p* = 0.26). However, social media addiction has significant indirect inverse effects on academic engagement. These effects are mediated by symptoms of depression (*β* = −0.15, *p* < 0.001) and by self-esteem (*β* = −0.06, *p* = 0.01). In general terms, addiction to social media increases the symptoms of depression (*β* = −0.44, p < 0.001), which in turn decreases academic engagement scores (*β* = −0.33, *p* < 0.001). Similarly, social media addiction decreases self-esteem (*β* = −0.29, *p* < 0.001), which serves as a factor that significantly increases engagement (*β* = 0.21, *p* < 0.01). On the other hand, addiction to social media increases anxiety symptoms (*β* = 0.42, *p* < 0.001), but these do not influence academic engagement (*β* = 0.10, *p* = 0.20); see Table 5.

**Table 5:** Direct and indirect effects.

Type	Effect	Estimate	95 % *CI*		*β*	*z*	*p*
			Lower	Upper			
Indirect	SMA ⇒ depression ⇒ academic engagement	−0.30 (0.08)	−0.48	−0.15	−0.15	−3.58	< **.001**
	SMA⇒ anxiety ⇒ academic engagement	0.09 (0.07)	−0.05	0.23	0.04	1.25	0.21
	SMA ⇒ self-esteem ⇒ academic engagement	−0.12 (0.05)	−0.22	−0.04	−0.06	−2.59	**0.01**
Component	SMA⇒ depression	0.56 (0.05)	0.46	0.66	0.44	11.29	< **.001**
	Depression ⇒ academic engagement	−0.54 (0.14)	−0.82	−0.26	−0.33	−3.76	< **.001**
	SMA⇒ anxiety	0.42 (0.04)	0.33	0.50	0.42	9.80	< **.001**
	Anxiety ⇒ academic engagement	0.21 (0.17)	−0.12	0.52	0.10	1.27	0.20
	SMA ⇒ self-esteem	−0.34 (0.05)	−0.44	−0.24	−0.29	−6.55	< **.001**
	Self-esteem ⇒ academic engagement	0.36 (0.11)	0.13	0.58	0.21	3.13	< **0.01**
Direct	SMA⇒ academic engagement	0.12 (0.11)	−0.09	0.36	0.06	1.13	0.26
Total	SMA ⇒ academic engagement	−0.21 (0.10)	−0.41	−0.01	−0.10	−2.08	**0.04**

*Note.* Confidence intervals were biased-corrected; Bootstrap was set at 10,000 replications. Significant *p*-values (<0.05) are presented in bold. SMA=Social Media Addiction.

## 4 Discussion

The theoretical contributions of this study shed light on the intricate interplay between social media addiction, mental health factors, and academic engagement among university students. By examining the mediating roles of self-esteem, symptoms of depression, and anxiety, the research provides a nuanced understanding of the indirect pathways through which social media addiction influences academic outcomes. Notably, the study highlights the non-linear relationship between social media addiction and academic engagement, emphasizing the importance of considering mediating factors in this complex relationship.

The main findings indicate that social media addiction is highly prevalent in this student population, with an average of 4.83 different social networks used at least once a week. Instagram and TikTok users reported significantly higher levels of social media addiction, symptoms of depression, and anxiety compared to non-users. These results are similar to those of other studies that indicate that Instagram and TikTok users are at particular risk of social media addiction [36,59]. The combination of engaging visual content, and the ability to interact with friends and followers through comments and likes will make it possible to create a powerful reward loop that makes it difficult for users to resist the temptation to check their accounts frequently [60]. In addition, constant social pressure to get more followers and likes increases stress levels, anxiety, and the risk of addiction. It is essential to consider that addiction to social media negatively affects mental health and productivity, increasing procrastination [61,62]. The identification of Instagram and TikTok users as particularly vulnerable to social media addiction, depression, and anxiety provides a nuanced and specific dimension to the ongoing discourse on the subject.

Contrary to initial expectations, the study revealed that social media addiction does not directly influence academic engagement scores. However, our findings suggest that social media addiction may contribute to academic disengagement by lowering self-esteem and elevating symptoms of depression. Low self-esteem can erode students' confidence in their capabilities and diminish their motivation to participate in academic endeavors. Furthermore, social media addiction is known to have a detrimental impact on self-esteem, which plays a significant role in bolstering academic engagement. As a result, numerous studies have provided evidence that social media addiction influences both self-esteem and academic performance [34,47].

Additionally, our findings indicate that social media addiction increases symptoms of depression and anxiety. These results are consistent with previous studies, which show that addiction to social media increases the risk of suffering depressive symptoms and anxiety [42,63]. Depression can lead to a loss of motivation, decreased concentration, and difficulty completing tasks, all of which can negatively impact academic performance. Then, as indicated by the data of our study, these symptoms of depression negatively impact the academic engagement of students, a finding consistent with previous research [64].

In terms of practical implications, the study offers valuable insights for addressing the impact of social media addiction on academic engagement in educational institutions. Recognizing the heightened vulnerability of Instagram and TikTok users, targeted awareness campaigns and support programs tailored to these platforms can be designed by educational institutions. Moreover, the identification of self-esteem as a mediating factor opens avenues for interventions, including counseling services and self-esteem enhancement programs, to alleviate the adverse effects of social media addiction on mental health and academic outcomes. This study provides actionable guidance for educators, mental health professionals, and policymakers to develop comprehensive approaches that foster positive academic engagement while effectively addressing the challenges associated with social media addiction among university students.

In this context, controlled and targeted use of social media emerges as a potentially constructive tool in higher education. Interactive virtual spaces, for instance, facilitate collaborative learning by improving communication between students and teachers, thereby enhancing engagement and academic performance [65]. Similarly, social media is a powerful channel for campaigns promoting mental health awareness, allowing users access to information and the opportunity to connect with professionals in the field [66]. These insights highlight the potential positive impact of judiciously leveraging social media within the higher education landscape.

Methodologically, our study provides a valuable framework for future research to investigate similar relationships in different contexts or populations, providing a standardized means of comparison. Furthermore, the questionnaires used in the study may serve as valuable tools for assessing the effectiveness of interventions aimed at mitigating the adverse effects of social media addiction on mental health and academic outcomes.

Despite the value of our findings, the study has limitations that must be considered. For instance, the non-probabilistic sample selection limits the generalizability of the results. Additionally, the study's cross-sectional design offers only a momentary glimpse into the relationship between the variables included in the study without the ability to track changes or trends over time. While the mediation model suggests a direction of influence from social media addiction to academic engagement via self-esteem and depressive symptoms, it is challenging to establish directionality in psychological studies definitively. The complex interplay between these variables could involve bidirectional or reciprocal relationships, which our model does not account for. Furthermore, the data was gathered during the COVID-19 pandemic, marked by heightened social media usage [38]. This specific context might restrict the applicability of the results to different periods.

To the best of the authors' knowledge, this study is the sole published research conducted within the Honduran context on this particular topic. As such, it constitutes a valuable contribution to guide future investigations. Subsequent studies should explore the subject more comprehensively through a qualitative approach, delving deeper into the analysis of various aspects. These aspects should encompass academic performance, repetition rates, absenteeism, university student attrition, and the advantages of integrating social media into higher education.

## Ethics statement

The research project was officially submitted to the Committee on Research Ethics (CRE) affiliated with the Faculty of Social Sciences at the National Autonomous University of Honduras. The CRE granted an Exemption Letter based on several factors, including 1) the study's observational design, 2) the anonymous data collection process, 3) the involvement of consenting individuals in the research, and 4) the low-risk nature of the study.

## Data availability statement

Data will be made available on request.

## CRediT authorship contribution statement

**Miguel Landa-Blanco:** Writing – review & editing, Writing – original draft, Visualization, Validation, Supervision, Project administration, Methodology, Investigation, Formal analysis, Data curation, Conceptualization. **Yarell Reyes García:** Writing – review & editing, Writing – original draft, Methodology, Investigation, Formal analysis, Conceptualization. **Ana Lucía Landa-Blanco:** Writing – review & editing, Writing – original draft, Investigation, Formal analysis. **Antonio Cortés-Ramos:** Writing – review & editing, Writing – original draft, Investigation, Formal analysis. **Eddy Paz-Maldonado:** Writing – review & editing, Writing – original draft, Investigation, Formal analysis.

## Declaration of competing interest

The authors declare that they have no known competing financial interests or personal relationships that could have appeared to influence the work reported in this paper.
